# Proteomic analysis of small acid soluble proteins in the spore core of *Bacillus subtilis ΔprpE *and 168 strains with predictions of peptides liquid chromatography retention times as an additional tool in protein identification

**DOI:** 10.1186/1477-5956-8-60

**Published:** 2010-11-22

**Authors:** Katarzyna Macur, Caterina Temporini, Gabriella Massolini, Jolanta Grzenkowicz-Wydra, Michał Obuchowski, Tomasz Bączek

**Affiliations:** 1 Department of Pharmaceutical Chemistry, Faculty of Pharmacy, Medical University of Gdańsk, Hallera 107, 80-416 Gdańsk, Poland; 2 Department of Pharmaceutical Chemistry, University of Pavia, Via Taramelli, 1227100 Pavia, Italy; 3 Pomeranian Science and Technology Park, Gdynia Innovation Centre, Zwycięstwa 96/9881-451 Gdynia, Poland; 4 Laboratory of Molecular Bacteriology, Department of Medical Biotechnology, Intercollegiate Faculty of Biotechnology, Medical University of Gdańsk, Gdańsk, Poland

## Abstract

**Background:**

Sporulation, characteristic for some bacteria such as *Bacillus subtilis*, has not been entirely defined yet. Protein phosphatase E (PrpE) and small, acid soluble spore proteins (SASPs) influence this process. Nevertheless, direct result of PrpE interaction on SASPs content in spore coat of *B. subtilis *has not been evidenced so far. As proteomic approach enables global analysis of occurring proteins, therefore it was chosen in this experiment to compare SASPs occurrence in two strains of *B. subtilis*, standard 168 and *ΔprpE*, lacking PrpE phosphatase. Proteomic analysis is still a challenge, and despite of big approach in mass spectrometry (MS) field, the identification reliability remains unsatisfactory. Therefore there is a rising interest in new methods, particularly bioinformatic tools that would harden protein identification. Most of currently applied algorithms are based on MS-data. Information from separation steps is not still in routine usage, even though they also provide valuable facts about analyzed structures. The aim of this research was to apply a model for peptides retention times prediction, based on quantitative structure-retention relationships (QSRR) in SASPs analysis, obtained from two strains of *B. subtilis *proteome digests after separation and identification of the peptides by LC-ESI-MS/MS. The QSRR approach was applied as the additional constraint in proteomic research verifying results of MS/MS ion search and confirming the correctness of the peptides identifications along with the indication of the potential false positives and false negatives.

**Results:**

In both strains of *B. subtilis*, peptides characteristic for SASPs were found, however their identification confidence varied. According to the MS identity parameter X_corr _and difference between predicted and experimental retention times (Δt_R_) four groups could be distinguished: correctly and incorrectly identified, potential false positives and false negatives. The *ΔprpE *strain was characterized by much higher amount of SASPs peptides than standard 168 and their identification confidence was, mostly for alpha- and beta-type SASP, satisfactory.

**Conclusions:**

The QSRR-based model for predicting retention times of the peptides, was a useful additional to MS tool, enhancing protein identification. Higher content of SASPs in strain lacking PrpE phosphatase suggests that this enzyme may influence their occurrence in the spores, lowering levels of these proteins.

## Background

Although bacterial endospores have been studied for over 130 years, there are still questions about basic mechanisms of their unique features, i.e. high resistance to environmental stress, such as high temperatures, chemicals or radiation, which enable them long term survival in unfavorable conditions [1]. *Bacillus subtilis *(*B. subtilis*) is a sporulating, model organism often used in the biochemical, genetic and molecular research, concerning Gram positive bacteria. Its sporulation process is very complicated and requires space and time gene expression regulation. About 25% of genes in chromosome of this bacterium are involved in spore formation. What is more, there are already 154 proteins identified, characteristic exclusively for spores, which do not occur in vegetative cells [2]. Small acid soluble spore proteins (SASPs) have been evidenced to be one of specific spore components, which may have an influence on their resistance to unfavorable conditions [3]. The SASPs belong to a group of at least sixteen proteins found in the core of spores produced by *B. subtilis *[4-6]. Genes coding for those proteins are expressed only during late steps of sporulation, mainly in the forespore compartment under the control of sigma G RNA polymerase subunit [6]. Its major role is to bind to chromosomal DNA and convert into A form. This is unique property of SspA (alpha-type) and SspB (beta-type) to promote conformational change in DNA in aqueous solution [7]. The result of such conversion is the increase of UV-resistance of spores. The major SASP are SspA and SspB (known also as alpha/beta-type SASPs) which may constitute 80% of all. The third major SASP is SspE protein (gamma-type). In contrast to alpha- and beta-type SASP, this protein exhibits only little homology among bacteria [8]. Moreover, it was previously shown that SspE protein does not bind to the chromosomal DNA and it was postulated that the protein has different physiological role, not identified yet [9]. It was also noticed that deletion of gene coding for one of α/β SASP leads to severe decrease of UV resistance of spores [10].

Protein phosphatase E (PrpE) is an enzyme of 27 kDa size, having in its structure motives characteristic for PPP protein phosphatases and diadenosinepolyphosphate hydrolases. PrpE phosphatase is a cytoplasmatic protein, present in a vegetative cell at a very small level as well as inside spores, in soluble and insoluble fractions. Some changes in spore coat of strain, which does not produce the functional PrpE protein, have been observed too. The research on the PrpE revealed that this enzyme is somehow implicated in expression of GerA germination receptors during sporulation, also directed by RNA polymerase with sigma G subunit [11]. This led to assume that lack of PrpE may also influence the expression of SASP.

To globally compare the occurrence of SASPs in standard 168 strain of *B. subtilis *and the one, lacking PrpE phosphatase, proteomic approach was chosen. However, despite of fast development of analytical tools in proteomics, identification and hence quantification of proteins and peptides, present in complex proteomic samples, still remains a challenge. Due to the great diversification and dynamic concentrations ranges of occurring peptides, one of the necessary steps in proteomic analysis is protein and peptides separation. Among many techniques, two-dimensional gel electrophoresis, liquid chromatography and capillary electrophoresis are used most frequently. The protein identification is then performed using mass spectrometry [12,13]. Huge amount of data coming from mass spectrometry analysis require bioinformatic tools to draw out right conclusions about the proteins presence and their concentrations in an analyzed sample. In general, many available database search algorithms, such as Mascot [14] or Sequest [15,16], identify analyzed protein samples by finding the best match between experimental spectra and theoretical ones, obtained for a set of possibly occurring peptides. True and false identifications are then distinguished by applying certain level of scoring threshold. However, in many cases, the confidence of identification is still unsatisfactory. It raises question if, on one hand, using high scoring criteria in filtering MS/MS spectra to lower the false discovery rate, the proteins that are really present in the sample, are not misidentified, or on the other hand, if lower them too much will not give untrue results. Therefore, there has been a raising interest observed in finding additional solutions lately, which may increase the identification reliability in proteomics. It is especially important in case of proteins that occur in low concentrations, and so are difficult to detect. Nevertheless they may remarkably influence the cell metabolism and for example may be used as biomarkers of certain diseases or help understanding biological processes. There are various approaches, aiming to raise protein identity, which use the mass spectrometry data [17,18]. One of most often applied strategies is Target-decoy approach [19]. There are also strategies, which additionally use information from separation step, for example from liquid chromatography, commonly combined on-line with mass spectrometer. A raising interest in application of peptides retention times prediction to protein identification in proteomics is recently observed [20-24]. Several ideas for predicting peptides retention times such as artificial neural networks [25,26] or regression models [27,28] are used. In case of multivariate modeling, the quantitative structure-retention relationships (QSRR) are often applied to predict the retention times of the test set of analyzed samples basing on the data from the model set. They are derived using statistic methods, relationships between chromatographic parameters and descriptors characterizing molecular structure of the analytes [29-31]. In this experiment a quantitative structure-retention relationships (QSRR) approach in multiple linear regression model (MLR) was used to build a model for predicting peptides retention times. The predicted (t_R_pred) and experimental (t_R_exp) retention times were then compared, and depending on the difference between them, the properly or improperly identified peptides were determined.

The aim of this project was to perform proteomic analysis of changes in small acid soluble proteins composition of spore coat produced by *B. subtilis *strain, lacking PrpE phosphatase, in comparison to standard 168, with the use of QSRR-based approach to predict peptides retention times and apply them as additional to MS/MS ion search parameter enhancing confidence of protein identification.

## Materials and methods

### Chemicals

Seven standard amino acids (isoleucine, leucine, methionine, phenylalanine, tryptophan, tyrosine, valine) were purchased from Fluka BioChemica (Buchs, Switzerland). Standard proteins (model proteins): bovine serum albumin (BSA), chicken egg ovalbumin (CEO), bovine milk lactoglobulin (BML), bovine milk β-casein (BMC), bovine myoglobin (BM), human serum albumin (HSA) and ribonuclease B (RibB); trypsin (Proteomic Grade), dithiothreitol (DTT), trifluoroacetic acid (TFA), ammonium bicarbonate and acetonitrile (ACN) (MS-grade) were obtained from Sigma-Aldrich (Steinheim, Germany). One of the model proteins, insulin-like growth factor-binding protein 1 (IGFBP-1), was purified from human amniotic fluid following in a previously reported procedure [32]. Water used in the experiments was deionized by passing through a Direct-Q™ (Millipore) system (Millipore, Bedford, MA, USA).

### Standard amino acids and model proteins solutions preparation

The standard amino acids solutions were prepared by dissolving in 0.1% aqueous solution of trifluoroacetic acid (TFA) (about 0.6 mg/mL). The solutions of standard proteins were obtained by dissolving the lyophilized standard proteins in deionized water (about 3 mg/mL). Then samples were treated as it is shown below in digestion protocol.

### Bacillus subtilis sample preparation

The growth conditions of *Bacillus subtilis *strains 168 and *ΔprpE*, spore purification and protein extraction procedures were as previously described [33]. After that, the samples were treated according to the below presented digestion protocol.

### Digestion protocol

To 1 mL of each model protein sample (~3 mg/mL), 300 μL of DTT (100 mM, freshly prepared in 100 mM ammonium bicarbonate buffer, pH 8.5) was added. The samples were kept in 60°C for 30 min, to enable the disulfide bridges reduction. After that, to each sample, 50 μg of trypsin was added (ratio 1:50 E/S). They were digested for 12 hours (overnight digestion) at 37°C. Then 0.1 mL of TFA was added to each sample to stop the digestion. The standard solutions concentrations were about 50 pmol/μL.

150 μL of DTT (100 mM, freshly prepared in 100 mM ammonium bicarbonate buffer, pH 8.5) was added to 1 mL of *Bacillus subtilis *spore cells lysates (1.2-1.5 mg/mL). The samples were stored in 60°C for 30 min, to allow reduction of the disulfide bridges. Next, 25 μg of trypsin was added (ratio 1:50 E/S) to each sample, which were digested for 12 hours (overnight digestion) at 37°C. Then 0.05 mL of TFA was added to each sample to stop the digestion. Received standard solutions concentrations were about 50 pmol/μL.

Tryptic digests were stored at -20°C (in this reaction mixture the disulfide bonds would not reoxidase if frozen). The LC-ESI-MS/MS analyses were carried out in three weeks at the latest (the shelf life of such frozen solution is couple of months) [34].

### LC conditions

The LC-MS apparatus was equipped with surveyor autosampler controlled at 20°C and thermostated column oven (Thermo Finnigan, San Jose, CA, USA), a quaternary gradient Surveyor MS pump (Thermo Finnigan, San Jose, CA, USA) with a diode array detection (DAD) system, and LTQ linear ion trap MS system with ESI ion source controlled by Xcalibur software 1.4 (Thermo Finnigan, San Jose, CA, USA).

The chromatographic separation was performed on C-18 analytical column: XTerra MS C18 3.5 μm (2.1 × 100 mm) column (Waters, Milford, MA, USA).

The mobile phase consisted of two solvents (A and B) mixed on-line. Solvent A was 0.1% aqueous solution of TFA and solvent B was ACN containing 0.1% TFA. The linear 90 min gradient time, from 0% B to 60% B, was applied. The flow rate was 200 μL/min. The injection volume was 10 μL.

### MS conditions

The MS/MS analysis was performed on Finnigan LTQ instrument (Thermo Finnigan, San Jose, CA, USA). The constant instrumental conditions, applied to generate mass spectra in positive ion mode, were as following: source voltage 4.62 kV, capillary voltage 40.97 V, sheath gas flow rate 39.99 (arbitrary units), auxiliary gas flow 10 (arbitrary units), sweep gas flow 0.95 (arbitrary units), capillary temperature 219.96°C, tube lens voltage 250.43 V. The collision-induced dissociation in the linear ion trap was used to generate MS/MS spectra. They were performed with an isolation width 3 Da (*m/z*), the activation amplitude was 35% of ejection RF amplitude, which corresponds to 1.58 V.

### Protein identification

The peptides *m/z *values were measured manually for the most intense peaks in acquired MS/MS spectra and automatically searched against the protein database (*fasta, downloaded from Expasy [35]) with the use of the Sequest Algorithm, included in Bioworks 3.0 (Thermo Finningan, San Jose, CA, USA). Experimental retention times (*t_R exp_*) of the analyzed peptides were defined at peak intensity maximum. Washburn *et al. *[36] filtering criteria were employed in the interpretation of the results obtained after the correlation analysis done on the peptides' experimental and the predicted retention times. The spectra for singly charged peptides with a cross-correlation score to a tryptic peptide (X_corr_) higher than 1.9, for doubly charged tryptic peptides with X_corr _over 2.2 and the spectra for triply charged tryptic peptides with X_corr _of at least 3.75 were accepted as correctly identified using Sequest software. All the analyzed spectra were characterized by ΔC_n _values above 0.08.

### QSRR analysis

The structural descriptors: logarithm of sum of retention factors of amino acids building certain peptide increased with one (*log Sum (k+1)_AA_*) and a calculated logarithm of n-octanol-water partition coefficient (*c log P*) of the analyzed peptides from investigated, standard proteins and *B. subtilis *cell lizates were calculated. The *log Sum (k+1)_AA _*descriptor was calculated using retention data for 7 the most retained amino acids (isoleucine, leucine, methionine, phenylalanine, tryptophan, tyrosine and valine). The k values for other, hardly retained amino acids, were ascribed (k = 0) and one was added to avoid zero in the calculation of the logarithm [37]. The *c *log *P *values were calculated applying average log P module in ALOGPS 2.1 software http://www.vcclab.org.

Then, multiple linear regression equation for model set of peptides based on the experimental retention times was derived using Microsoft Excel software (Microsoft Co., Redmond, WA, USA) and Statistica (StatSoft, Tulsa, OK, USA) run on a personal computer. Regression coefficients (± standard deviations), multiple correlation coefficients, R, standard errors of estimate, s, significance levels of each term and of the whole equations, p, and values of the F-test of significance, (F) were calculated. The general form of QSRR equation to predict peptides retention times is:

(1) $$t_{R}=k_{1}+k_{2}log\;Sum{\left(k+1\right)}_{AA}+k_{3}clog\;P$$

where *t_R _*is the gradient HPLC retention time and *k_1_*-*k_3 _*are regression coefficients.

Finally, the following general equation, with a satisfactory statistical quality, was derived:

(2) $$t_{R}=-\text{25}.0\text{7}\left(\pm \right)+\text{33}.\text{16}\left(\pm \right)log\;Sum{\left(k+1\right)}_{AA}+0.\text{6}0\left(\pm \right)clog\;P$$

*p *= 4×10^-15^, *p *= 9×10^-32^, *p *= 7×10^-10^,

with *n *= 50, *R *= 0.974, *s *= 1.45, *F *= 431, *p *< 6×10^-31^.

It was obtained using the model set consisted of 50 peptides (Table 1) and verified with a test set of 21 peptides. They were identified by LC-ESI-MS/MS analysis of 8 model proteins [36]. Due to the fact that X_corr _values are used as indication of proper or improper match between theoretical and experimental spectra, the model set included peptides with the best identification reliability, i.e. with the highest X_corr _score, whereas for test peptides these values were lower, but still qualifying them to correctly identified ones.

**Table 1:** Peptides from model proteins used to derive the QSRR model.

Model proteins										
**Protein**	**Peptide sequence**	**m/z**	**Charge**	**X**_**corr**_	**Missed cleavages**	***c*log*P***	**log Sum (k+1)**_**AA**_	**t**_**R**_**exp**	**t**_**R**_**pred**	**Δt**_**R**_

BML	ALKALPMHIR	575.7332	2	3.06	1	-1.74	1.3542	25.12	25.13	0.01
BSA	LFTFHADICTLPDTEKQIK	1111.2810	2	3.21	1	-4.60	1.6674	32.60	32.62	0.02
COA	KIKVYLPR	509.1467	2	2.27	2	-0.95	1.3005	24.06	24.08	0.02
HSA	LVNEVTEFAK	575.6500	2	3.42	0	-2.44	1.3540	24.53	24.56	0.03
BML	YTRKVPQVSTPTLVEVSR	1031.1858	2	3.01	2	-5.67	1.4758	25.80	25.77	0.03
IGFBP-1	ALHVTNIK	896.0698	1	2.48	0	-3.14	1.2148	19.69	19.64	0.04
BSA	DTHKSEIAHR	597.6416	2	2.80	1	-7.77	1.1246	13.15	13.10	0.05
HSA	AAFTECCQAADK	687.7000	2	3.07	0	-5.69	1.2657	19.31	19.18	0.13
IGFBP-1	ALPGEQQPLHALTR	766.3710	2	3.37	0	-5.22	1.4145	24.36	24.21	0.15
BMC	VKEAMAPK	874.0844	1	2.13	1	-2.19	1.0165	14.40	14.21	0.19
RibB	QHMDSSTSAASSSNYCNQMMK	789.8418	3	4.75	0	-11.80	1.4546	20.46	20.18	0.28
BSA	AFDEKLFTFHADICTLPDTEK	814.9098	3	5.11	1	-4.51	1.7127	34.45	34.11	0.34
RibB	HIIVACEGNPYVPVHFDASV	1113.7305	2	5.15	0	-3.51	1.6128	32.15	31.79	0.36
HSA	VHTECCHGDLLECADDRADLAK	1295.3400	2	4.47	1	-9.95	1.5447	24.98	24.48	0.50
BSA	AFDEKLFTFHADICTLPDTEKQIK	1406.5952	2	4.42	2	-4.95	1.7629	34.77	35.32	0.55
HSA	DLGEENFK	952.0000	1	2.09	0	-4.48	1.2654	19.46	20.15	0.69
HSA	CCAAADPHECYAK	778.7900	2	3.46	0	-5.77	1.2195	16.98	17.68	0.70
IGFBP-1	IPGSPEIR	869.0012	1	2.18	0	-2.43	1.1657	19.40	18.68	0.72
BML	LKPDPNTLCDEFKADEKKFWGKYLYEIAR	1174.0045	3	4.49	5	-5.45	1.8691	39.20	38.24	0.96
IGFBP-1	ALHVTNIKK	1024.2427	1	2.01	1	-4.64	1.2405	18.27	19.24	0.97
BMC	VLPVPQKAVPYPQR	796.9551	2	3.32	1	-3.24	1.3948	24.20	25.19	0.99
HSA	AEFAEVSK	880.9700	1	2.10	0	-2.94	1.1909	18.06	19.06	1.00
IGFBP-1	ALHVTNIKK	512.6214	2	2.70	1	-4.64	1.2405	18.24	19.24	1.00
HSA	TCVADESAENCDK	751.2300	2	4.05	0	-7.23	1.1488	15.32	14.29	1.03
HSA	NECFLQHK	1077.1600	1	2.33	0	-3.74	1.2654	19.67	20.74	1.07
BSA	TCVADESHAGCEK	675.7310	2	3.47	0	-7.76	1.1488	14.99	13.87	1.12
BSA	CASIQKFGER	570.1555	2	2.78	1	-4.66	1.2958	19.81	20.95	1.14
HSA	VHTECCHGDLLECADDR	1046.0500	2	6.23	0	-8.42	1.4161	22.86	21.69	1.17
BSA	LFTFHADICTLPDTEK	926.5506	2	4.79	0	-3.25	1.6039	32.90	31.72	1.18
IGFBP-1	RIPGSPEIR	513.0938	2	2.63	1	-3.50	1.1944	20.00	18.72	1.29
BSA	HLVDEPQNLIK	653.7467	2	3.48	0	-4.68	1.3690	24.56	23.23	1.33
BSA	TCVADESHAGCEKSLHTLFGDELCK	1348.0020	2	3.77	1	-7.37	1.6483	31.14	29.79	1.35
RibB	YPNCAYK	916.9852	1	2.58	0	-2.34	1.1508	16.78	18.29	1.51
BML	LRCASIQKFGER	704.8278	2	3.82	2	-4.96	1.4107	22.79	24.31	1.51
IGFBP-1	WKEPCRIELYR	747.3795	2	2.70	2	-3.52	1.4991	26.67	28.23	1.56
BML	TPEVDDEALEKFDK	818.8696	2	5.09	1	-6.12	1.4067	24.94	23.25	1.69
HSA	LDELRDEGK	538.0800	2	2.52	1	-5.34	1.2299	16.62	18.35	1.73
HSA	YICENQDTISSKL	732.6700	2	3.80	1	-5.93	1.4238	22.69	23.93	1.24
BSA	YICDNQDTISSKLK	814.9150	2	4.03	1	-6.10	1.4504	22.75	24.63	1.88
BSA	QTALVELLKHKPK	753.4154	2	2.89	2	-3.08	1.4159	27.89	25.98	1.91
BMC	VKEAMAPKHK	570.1986	2	2.90	2	-3.41	1.0930	13.48	15.62	2.14
COA	ELINSWVESQTNGIIR	930.5307	2	3.93	0	-5.86	1.6097	31.95	29.80	2.15
COA	ISQAVHAAHAEINEAGR	887.9599	2	3.19	0	-7.56	1.3932	19.51	21.67	2.16
BSA	YICDNQDTISSK	694.2494	2	3.80	0	-6.61	1.3468	18.79	20.98	2.19
BML	SHCIAEVEKDAIPENLPPLTADFAEDKDVCK	1133.9316	3	5.65	2	-3.53	1.7342	33.13	35.57	2.44
COA	GGLEPINFQTAADQAR	844.9119	2	4.19	0	-6.71	1.4735	27.41	24.86	2.55
BMC	EAMAPKHKEMPFPK	821.4917	2	3.87	2	-3.31	1.3625	21.52	24.12	2.60
IGFBP-1	FYLPNCNKNGFYHSR	931.0381	2	2.76	0	-5.83	1.5912	26.57	29.25	2.68
HSA	YICENQDSISSK	723.2500	2	4.04	0	-6.77	1.3468	18.06	20.85	2.79
BSA	SLHTLFGDELCK	682.2826	2	3.55	0	-3.32	1.4829	30.69	27.88	2.81

## Results and discussion

A statistically reliable QSRR equation (Equation 2), derived using a model set of peptides from 8 model proteins, was applied to predict retention times of peptides from group of small acid soluble proteins from proteomes of *Bacillus subtilis *strains *ΔprpE *and 168. The data were then analyzed together with usage of Sequest software with above mentioned threshold level.

The gradient retention time prediction of analyzed peptides, originated from several types of small, acid soluble spore proteins (SASPs) from proteomic samples of both analyzed strains of *Bacillus subtilis*, enabled distinguishing them into four groups, depending on their identification confidence (Table 2 and Table 3). In the first group there were peptides identified with high X_corr _values and differences between their experimental (t_R_exp) and predicted (t_R_pred) retention times (Δt_R_) lower than 5 minutes (from 0.01 to 3.90 min). It can be noticed that small differences between predicted and experimental retention times correspond with proper determination level of peptide presence in analyzed sample. The second group consisted of peptides, which identification reliability was poor according to their X_corr _values, and their differences between predicted and experimental retention times were characterized by high values as well (from 5.66 up to even 83.74 min in 90 min run). In this case, detailed comparison between MS and MS/MS spectra proved that the matches between theoretical and experimental ones were not good. However in different parts of chromatogram the parent ions of certain m/z values could be found, what indicated that they possibly originated from peptides of another sequences. Hence, it may be observed that low X_corr _scores correlate with big differences between predicted and experimental retention time of certain peptide, what additionally proofs their improper identification. Peptides from the third group were described by X_corr _values classifying them to correctly identified ones, but their Δt_R _were between 6.41 to 10.05 min. It may suggest that some of them could be potential false positives. Therefore further examination, whether they are present or not in analyzed samples, would be useful. The identification confidence of peptides from fourth group was insufficient, considering X_corr _scores, however their Δt_R _values were low (from 1.90 to 4.57 min). This may indicate that some of them could be potential false negatives. In this case additional experiment, proving their real occurrence in any of the analyzed strains of *B. subtilis*, could help to distinguish right and wrong identifications as well.

**Table 2:** The small, acid soluble spore proteins detected in Bacillus subtilis Δ prpE strain.

Δ*prp E*										
**Protein**	**Peptide sequence**	**m/z**	**Charge**	**X**_**corr**_	**Missed cleavages**	***c*log*P***	**log Sum(k+1)**_**AA**_	**t**_**R**_**exp**	**t**_**R**_**pred**	**Δt**_**R**_

**Proteins correctly identified according to X_corr _values and correctly according to Δt_R_:**										

SASP - alpha-type	LVSFAQQNMGGGQF	742.8313	2	4.72	0	-0.78	1.5218	28.39	30.85	-2.46
SASP - alpha-type	RLVSFAQQNMGGGQF	820.9245	2	3.69	1	-1.13	1.5346	30.98	30.99	-0.01
SASP - alpha-type	ANNNSGNSNNLLVPGAAQAIDQMK	814.8842	3	3.41	0	-1.15	1.5762	31.02	32.32	-1.30
SASP - alpha-type	ANGSVGGEITKRLVSFAQQNMGGGQF	1327.9721	2	3.84	2	-0.68	1.696	39.28	36.53	2.75
SASP - beta-type S	ANQNSSNDLLVPGAAQAIDQMK	1143.7547	2	3.25	0	-1.18	1.5526	31.64	31.54	0.10
SASP - beta-type S	LEIASEFGVNLGADTTSR	941.0171	2	4.30	0	-1.25	1.5661	33.14	31.92	1.22
SASP - *B. subtilis*	NVIQGALEDAGSALKDDPLQEAVQK	1305.9299	2	4.23	1	-1.01	1.628	37.99	34.09	3.90
SASP - *B. subtilis*	NVIQGALEDAGSALKDDPLQEAVQK	870.9532	3	3.80	1	-1.01	1.628	37.95	34.09	3.86

**Proteins incorrectly identified according to X_corr _values and incorrectly according to Δt_R_:**										

SASP - alpha/beta-type	GRRRGV	700.8170	1	0.69	3	-2.48	0.8503	91.69	7.95	83.74
SASP - alpha/beta-type	EQMKLEIAS	525.1123	2	0.85	1	-3.7	1.2459	26.26	19.75	6.51
SASP - gamma-type	AQQVR	601.6790	1	1.22	0	-1.95	0.7843	11.89	6.23	5.66
SASP - gamma-type	EFASE	582.5829	1	0.65	0	-2.58	1.0583	22.38	14.57	7.81
SASP - gamma-type	QNQQSAGQQGQFGTEFASETDAQQVR	1421.4511	2	1.90	0	-1.72	1.6016	24.73	32.70	-7.97
SASP - gamma-type	QQSAAGQGQFGTEFASETNAQQVRKQNQ	1014.0562	3	1.64	2	-1.43	1.6228	27.28	33.60	-6.32
SASP - gamma-type	KQNQQSAAGQGQFGTEFASETNAQQVRK	1014.0705	3	2.36	2	-1.27	1.6228	27.37	33.73	-6.36
SASP - *B. subtilis*	ALKDDPLQEAVQKKKNNR	1048.6888	2	0.78	4	-1.49	1.4324	37.98	27.43	10.55
SASP SspI	PGLGVLFEV	931.1109	1	0.78	0	0.81	1.408	3.28	28.40	-25.12
SASP Tlp	QNGYR	637.6682	1	0.54	0	-2.24	0.9333	91.35	10.80	80.55

**Potential false-positives: proteins correctly identified according to X_corr _values and incorrectly according to Δt_R_:**										

SASP - gamma-type	KQNQQSAGQQGQFGTEFASETDAQQVR	990.6917	3	6.97	1	-1.43	1.6123	23.22	33.27	-10.05
SASP - gamma-type	QQNQSAEQNKQQNS	816.8161	2	2.81	1	-2.74	1.1461	9.12	17.27	-8.15
SASP - gamma-type	KQNQQSAAGQGQFGTEFASETNAQQVR	971.3462	3	5.20	1	-1.33	1.6123	25.79	33.34	-7.55

SASP - gamma-type	KQNQQSAAGQGQFGTEFASETNAQQVR	1456.5194	2	5.75	1	-1.33	1.6123	25.84	33.34	-7.50
SASP - *B. subtilis*	VVVSVNTDQDQAQAQSQDGED	1117.6176	2	4.73	0	-2.47	1.4038	19.36	25.77	-6.41

**Potential false-negatives: proteins incorrectly identified according to X_corr _values and correctly according to Δt_R_:**										

SASP - beta-type S	ANGSVGGEITKR	595.1525	2	1.82	1	-1.75	1.2151	22.14	20.24	1.90
SASP - *B. subtilis*	NVIQGALEDAGSALKDDPLQEAVQKK	913.6775	3	2.36	2	-0.88	1.6381	38.31	34.52	3.79
SASP - *B. subtilis*	LTGGVTPQGDLEGNTHNDPKTELEER	936.9845	3	2.69	1	-1.59	1.5917	27.91	32.48	-4.57

**Table 3:** The small acid soluble spore proteins detected in Bacillus subtilis 168 strain.

168										
**Protein**	**Peptide sequence**	**m/z**	**Charge**	**X**_**corr**_	**Missed cleavages**	***c*log*P***	**log Sum(k+1)**_**AA**_	**t**_**R**_**exp**	**t**_**R**_**pred**	**Δt**_**R**_

**Proteins correctly identified according to X_corr _values and Δt_R_:**										

SASP - alpha-type	LVSFAQQNMGGGQF	742.8313	2	4.73	0	-0.78	1.5218	28.61	30.85	-2.24
SASP - beta-type S	ANGSVGGEITK	517.0593	2	2.40	0	-1.58	1.1878	16.22	19.49	-3.27

**Proteins incorrectly identified according to X_corr _values and Δt_R_:**										

SASP - alpha/beta-type	LEIASEFGVQLGAETTSR	637.0292	3	0.54	0	-1.08	1.5661	19.25	32.05	-12.80
SASP - alpha/beta-type	DLGFYDTVK	1058.1664	1	0.79	0	-2.79	1.3818	96.39	24.82	71.57
SASP - beta-type S	LVSFAQQQMGGR	1322.5195	1	0.65	0	-1.39	1.3947	81.37	26.30	55.07
SASP - beta-type S	LEIASEFGVNLGADTTSR	941.0171	2	2.07	0	-1.25	1.5661	40.37	31.92	8.45
SASP - gamma-type	QQNQSAEQNK	588.0960	2	0.84	0	-1.72	1	5.64	13.34	-7.70

**Potential false-negatives: proteins incorrectly identified according to X_corr _values and correctly according to Δt_R_:**										

SASP - *B. subtilis*	DAAVAK	574.6503	1	0.92	0	-2.75	0.8503	9.64	7.74	1.90

The *ΔprpE *strain was characterized by big amount of various peptides coming from SASPs of alpha-, beta- and gamma-type. There were also detected peptides, characteristic for SASPs, however it was not possible to distinguish definitely from which of them. Only alpha- and beta-type SASPs were identified with a satisfactory level of confidence according to X_corr _scores and their Δt_R_. In the incorrectly identified group were peptides typical for alpha/beta-type, gamma-type SASP, SspI and Ssp Tlp. In the group of potential false positives there were peptides characteristic for gamma-type SASP. Although the MS confirmation of their presence in the sample was good, the Δt_R _values were high, what may suggest, that they did not occur. One peptide of beta-type SASP and two from SASPs in general characterized with a low Δt_R _values, but their MS identification was insufficient for the applied threshold level. It may mean that these peptides were falsely classified to the improperly identified according to X_corr _score, and the proteins, for which they are typical, really were present in the analyzed sample (Table 1). In contrast, in standard 168 strain, lower amount of peptides from SASPs was detected. Moreover, only two peptides, from alpha- and beta-type SASPs, were identified correctly according to X_corr _scores and Δt_R _values as well. Most of the peptides, characteristic for alpha/beta-, beta- and gamma-type SASP were incorrectly identified considering their X_corr _and Δt_R _values, hence they were not present in the analyzed sample. There was only potential false negative peptide of a sequence typical for SASP, because, in spite of the fact that its X_corr _was low, Δt_R _value indicated that it was correctly identified. In this strain no potential false positives could be found (Table 2).

## Conclusions

Thanks to proteomic approach applied in this experiment, it was possible to analyze the whole protein content at once, what enabled easier distinction between both strains of *B. subtilis*: the standard 168 and the one lacking PrpE phosphatase (*ΔprpE*), in view of small, acid soluble spore proteins (SASPs) occurrence.

A QSRR-based retention time prediction model revealed to be a useful tool, supporting MS/MS ion search, in analysis of small acid soluble proteins (SASPs) from two *Bacillus subtilis *strains. The PrpE phosphatase lacking strain was characterized by the occurrence of alpha- and beta-type SASPs, which identification confidence was proved both with Sequest X_corr _values and small Δt_R_. The gamma-type SASP proved to occur in the *ΔprpE *strain, however the Δt_R _values suggest, that it might be potentially false positive identified protein. The wild 168 strain was characterized by poor content of SASPs and, moreover, they were identified basing only on one peptide occurrence, hence, according to proteomic standards, may not be really present. This suggests that the absence of PrpE phosphatase results in higher amount of SASPs, especially alpha- and beta-type, in the spores.

## Competing interests

The authors declare that they have no competing interests.

## Authors' contributions

JG-W prepared *B. subtilis *samples. KM and CT performed the LC-ESI-MS/MS analysis. TB and KM performed QSRR-based MLR analysis. GM and TB were coordinating the experiments. KM, CT, GM, JG-W, MO and TB, were co-working during results interpretation. All the authors contributed to the manuscript writing.
